# Performance of Ultrasound-Guided Core Biopsy Driven by FDG-avid Supraclavicular Lymph Nodes in Patients With Suspected Lung Cancer

**DOI:** 10.3389/fmed.2021.803500

**Published:** 2022-01-20

**Authors:** Tongtong Wang, Junbao Liu, Ningning Lv, Shi Xuan, Lin Bai, Bin Ji, Shi Gao

**Affiliations:** ^1^Department of Nuclear Medicine, China-Japan Union Hospital of Jilin University, Changchun, China; ^2^Department of Surgery, China-Japan Union Hospital of Jilin University, Changchun, China

**Keywords:** ultrasound-guided core biopsy, supraclavicular lymph nodes, lung cancer–diagnosis, FDG-PET/CT, performance

## Abstract

**Objectives:**

Ultrasound-guided core biopsy (UGCB) for supraclavicular lymph nodes (SLNs) represents an attractive procedure to obtain tissues for lung cancer confirmation. The aim of the present study is to evaluate the performance of UGCB driven by FDG-avid SLNs, as performed by nuclear medicine physicians, in patients with suspected lung cancer.

**Methods:**

Institutional database in our hospital was searched for eligible patients between September 2019 and March 2021. A 3-12 MHz linear probe was used to guide the biopsy process and to ensure that the needle tip was being directed at the metabolically active area that had been indicated by side-by-side PET/CT images. Diagnostic yield, malignancy rate, molecular testing results, and complications were reviewed.

**Results:**

Among the 54 patients included in this study, definite pathological diagnosis from UGCB specimens was achieved in 53 patients, reaching a diagnostic yield of 98.1% (53/54) and a malignancy rate of 96.2% (51/53). Among the 50 patients confirmed as lung cancer, thirty-eight were spared from further invasive procedures which had been planned. Molecular analyses were adequately performed on all the 38 specimens obtained from non-small cell lung cancer (NSCLS). The positive rate was 36.8% (14/38) for epidermal growth receptor (EGFR) mutation and 31.6% (12/38) for anaplastic lymphoma kinase (ALK) translocation. 28.9% (11/38) of the patients had a tumor proportion score (TPS) ≥ 50% for PD-L1 expression. No complication was observed and the average biopsy time was 15 min.

**Conclusions:**

Nuclear medicine physicians-performed UGCB driven by FDG-avid SLNs in suspected lung cancer patients could produce a high performance in terms of diagnostic yield, malignancy rate, and molecular analysis, which may obliviate more invasive interventional procedures and lead to fast decisions on subsequent management.

## Introduction

Ultrasound-guided core biopsy (UGCB) for supraclavicular lymph nodes (SLNs) represents an attractive technique to obtain tissues for lung cancer confirmation (1–3). It is a simple, rapid and minimally invasive choice that could avoid the risk of complications from more costly procedures such as bronchoscopy, CT-guided lung biopsy and diagnostic surgery. Besides, lung cancer confirmation through SLN tissues could directly upstage the patients into stage IIIB disease which would exclude surgical therapeutic options according to guidelines (4, 5).

In recent years, ^18^F-FDG-PET/CT has been increasingly used in the diagnostic workup of a pulmonary mass to stratify malignancy risk and provide possible staging information (6–8). The PET/CT findings may help to optimize UGCB for SLNs from several aspects. Firstly, it could reveal more suspicious SLNs as compared to initial examinations such as CT (9). This will render more choices in selecting SLNs with easier needle pass routes. Secondly, the PET scan could provide whole-body information of tumor involvement including easy-to-access superficial distant metastases, which may obliviate the need for sampling SLNs. Finally, but most importantly, biopsy driven by FDG-avid lesions have demonstrated a high diagnostic yield in various type of changes including thoracic, abdominal, bone, muscle, and breast lesions (10, 11). Because FDG avidness in malignant lesions represents the existence of metabolically active tumor cells, sampling of FDG-avid lesions will be more likely to yield representative material for molecular analysis (12, 13).

Nuclear medicine physicians collect comprehensive patient information and are the first to learn about the PET/CT results. If they become proficient in UGCB, the diagnostic pathway of lung cancer could be significantly optimized. Hence, we hypothesize that UGCB driven by FDG-avid SLNs in suspected lung cancer patients, as performed by nuclear medicine physicians, would produce high performance in terms of diagnostic yield, malignancy rate, molecular testing, and complications. In the present study, we tried to verify this hypothesis by retrospectively reviewing these data within our institution.

## Materials and Methods

### Patient Selection

Since September 2019, we began to routinely implement UGCB upon eligible patients with FDG-avid SLNs by simultaneously consulting side-by-side PET/CT images. Before this time point, WT and LN, which are nuclear medicine physicians in our department, had received over 5 years training for using ultrasound. The following conditions must be fulfilled in order to implement this procedure: (1) suspected lung cancer patients upon initial PET evaluation; (2) an accessible FDG-avid SLN was identified in the absence of other easy-to-access superficial distant metastases, as determined by WT and LN; (3) collective discussions among referring physicians, surgeons and nuclear medicine physicians decided to recommend this procedure to the patients; (4) patients agreed with receiving such an intervention upon recommendation including both the pros (it is simple, with less pain, and may lead to fast decision on subsequent management) and cons (it may not define the highest disease stage and information from SLN may not replace those of primary tumor in terms of molecular analysis). Between 2019 and March 2021, our department completed 866 PET scans for initial evaluation of suspected lung cancer patients. Among them, a total of 54 patients received nuclear medicine physicians-performed UGCB driven by FDG-avid SLNs.

This retrospective investigation based on institutional database was approved by the ethics committee of our institution.

### ^18^F-FDG-PET/CT

All patients underwent ^18^F-FDG-PET/CT within 2 days before UGCB. The PET/CT scans were acquired by using two dedicated diagnostic PET/CT devices uMI 510 unit and uMI 780 unit (United Imaging, Shanghai, China). All PET-CT acquisitions were performed 40–60 min following intravenous injection of 3.5 MBq/kg of ^18^F-FDG after a fasting period of at least 6 h. Integrated PET/CT images were corrected for scatter and attenuation based on CT information.

### Sampling and Histological Evaluation

**Figure 1:**
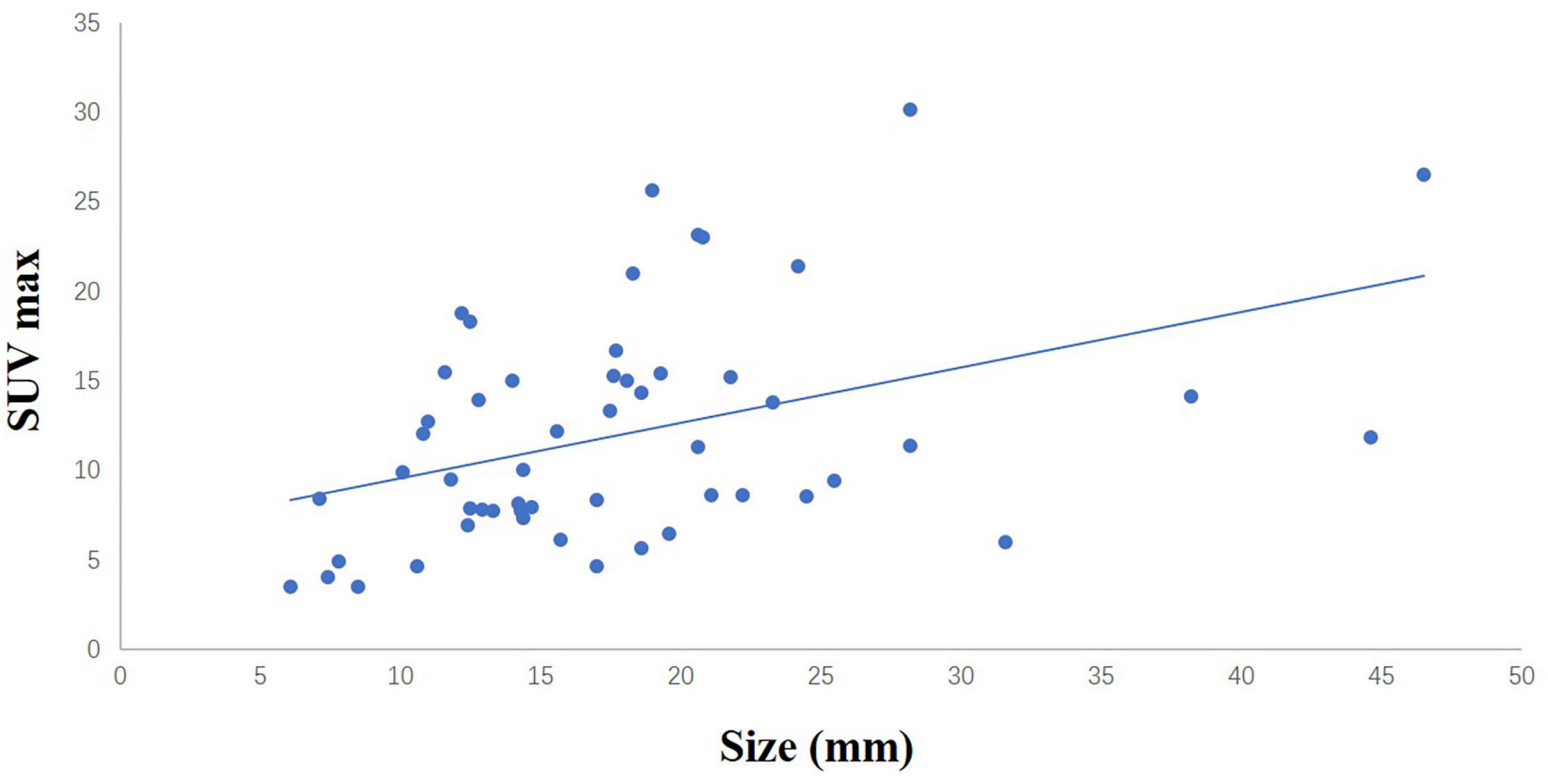
The liner relationship between the size of sampled SCLNs and SUV max (*r* = 0.416, *p* < 0.05).

**Figure 2:**
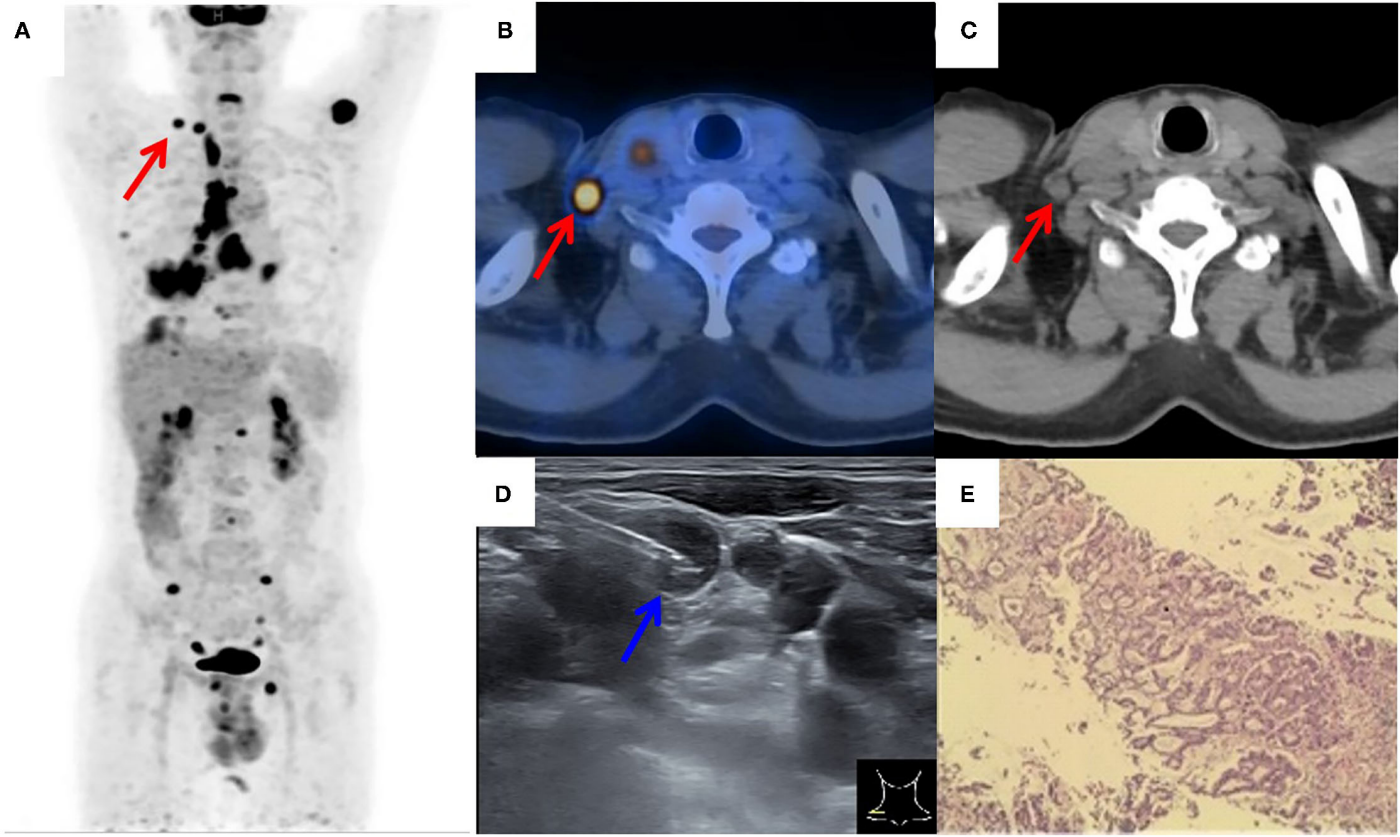
^18^F-FDG PET/CT of a patient with suspected lung cancer. **(A)** MIP image showed primary lung cancer in the right lower lobe with extensive right supraclavicular (red arrow), hilar, mediastinum, right pleura, liver, and multiple bone tracer accumulation; **(B,C)** axial PET/CT image showed an FDG-avid SLN (red arrow) on the left side; **(D)** by consulting side-by-side PET/CT image, FDG-avid SLN was successfully located by the needle (blue arrow) under real-time ultrasound guidance; **(E)** a lung adenocarcinoma was histopathologically diagnosed.

All FDG-avid SLNs were detectable on ultrasound. Patients were placed in supine position, with the shoulders raised to fully expose the neck for ultrasound scanning. Ultrasound images were evaluated side by side with the serial transverse PET/CT images (recorded by iPad) to identify the lesion or area of interest. Local anesthesia was achieved by injecting 5 mL of 2% lidocaine hydrochloride. 18-gauge biopsy needles (Argon Medical Devices, USA), with a diameter of 1 mm for the cutting needle and a length of 9 mm for the sampling notch were used to obtain SLN tissue. A 3–12 MHz linear probe (N1500, Neusoft, China) was used to guide the biopsy process and to ensure that the needle tip was being directed at the metabolically active area that had been indicated by the PET/CT images. An average of two to three passes were made to obtain sufficient tissue. PET/CT and ultrasound images in representative patients undergoing UGCB are shown in Figures 1, 2.

SLNs samples were subjected to hematoxylin and eosin (HE) staining and immunohistochemical staining for histological evaluation. For patients in whom the tissue diagnosis based on the SLN samples were not metastatic lung cancer, pathological results from the lung lesions were obtained.

### Molecular Analysis

When non-squamous cell lung cancer was diagnosed, the SLNs samples were assessed for molecular analysis. The test for epidermal growth receptor (EGFR) mutations was performed using the ADx-ARMS kit (Amplification Refractory Mutation System) that covers exons 18, 19, 20, and 21 (Amoy Diagnostics, Xiamen, China). The analysis for anaplastic lymphoma kinase (ALK) translocation was performed by immunohistochemistry (IHC, clone D5F3, VMSI) on formalin-fixed paraffin-embedded sections. PD-L1 immunohistochemistry was performed using the Dako 22C3 antibody clone (mouse monoclonal primary anti–PDL1 antibody, prediluted, clone 22C3, Dako) according to a previously described protocol (14). The level of PD-L1 expression was based on a tumor proportion score (TPS) which was defined as the percentage of viable tumor cells showing partial or complete membrane labeling regardless of intensity or completeness.

## Results

A total of 54 suspected lung cancer patients who have underwent UGCB driven by FDG-positive SLNs and were included in this retrospective study. Table 1 shows the clinical characteristics of our patient population. The median of age was 63 (range, 47–77) years. Thirty patients (55.6%) were males and 40 (74.1%) had a smoking history. The median of the size of sampled SLNs was 12.5 mm (range, 4.9–32.0 mm) in their short axis and 17.0 mm (range, 6.1–46.5 mm) in their long axis. The median of maximum standard uptake value (SUVmax) was 10.7 (range, 3.5–30.2). Figure 1 showed the linear relationship between the size of sampled SLNs and SUVmax (*r* = 0.416, *p* < 0.05). The most common histopathological diagnoses were adenocarcinoma (42, 77.8%).

**Table 1:** Patients' characteristic.

**Characteristic**	**Value**
Year	63 (47–77)
Sex	
Female	24 (44.4%)
Male	30 (55.6%)
SLN diameter on CT (mm)	
Long axis	17.9 (6.1–46.5)
Short axis	12.9 (4.9–32.0)
Smoking history	
Current	35 (64.8%)
Former	5 (9.3%)
Never	14 (25.9%)
Metastatic site	
Supraclavicular lymph node	52
Neck lymph node	21
Bone	18
Pleura	13
Liver	11
Brain	6

The diagnostic yield was 98.1% (53/54). There was only one non-diagnostic patient who was established as lung adenocarcinoma by subsequent guided bronchoscopy toward the lung lesion. The malignancy rate was 96.2% (51/53). The two benign SLNs revealed by UGCB were tuberculosis and hyperplasia. There is also one SLN of lymphoma and pathological results from the lung lesion confirmed this diagnosis.

Fifty patients were confirmed as lung cancer by UGCB. The suspected stage and metastatic sites of these patients were summarized in Table 2. Among them, 38 were spared from further bronchoscopy, CT-guided lung biopsy and diagnostic surgery which had already been planned.

**Table 2:** Results of tissue diagnosis.

**Tissue diagnosis**	**Number**
Adenocarcinoma	42 (77.7%)
Squamous cell carcinoma	3 (5.5%)
Small-cell lung cancer	5 (9.2%)
Lymphoma	1 (1.9%)
Tuberculosis	1 (1.9%)
Hyperplasia	1 (1.9%)
Non-diagnostic	1 (1.9%)

There were 45 patients with non-small cell lung cancer (NSCLC) in the present study. Thirty-eight of them received molecular analyses for SLNs specimens and they were all adequately performed. The positive rate was 36.8% (14/38) for EGFR mutation and 31.6% (12/38) for ALK translocation. In all these specimens, PDL1 testing was also successfully carried out, with PDL1 not expressed in 13/38 (34.2%), PDL1 >1% and <50% in 36.8% (14/38), and PDL1 > 50% in 11/38 (28.9%).

No complication was recorded in the present patient cohort. The average biopsy time was 15 min.

**Figure 3:**
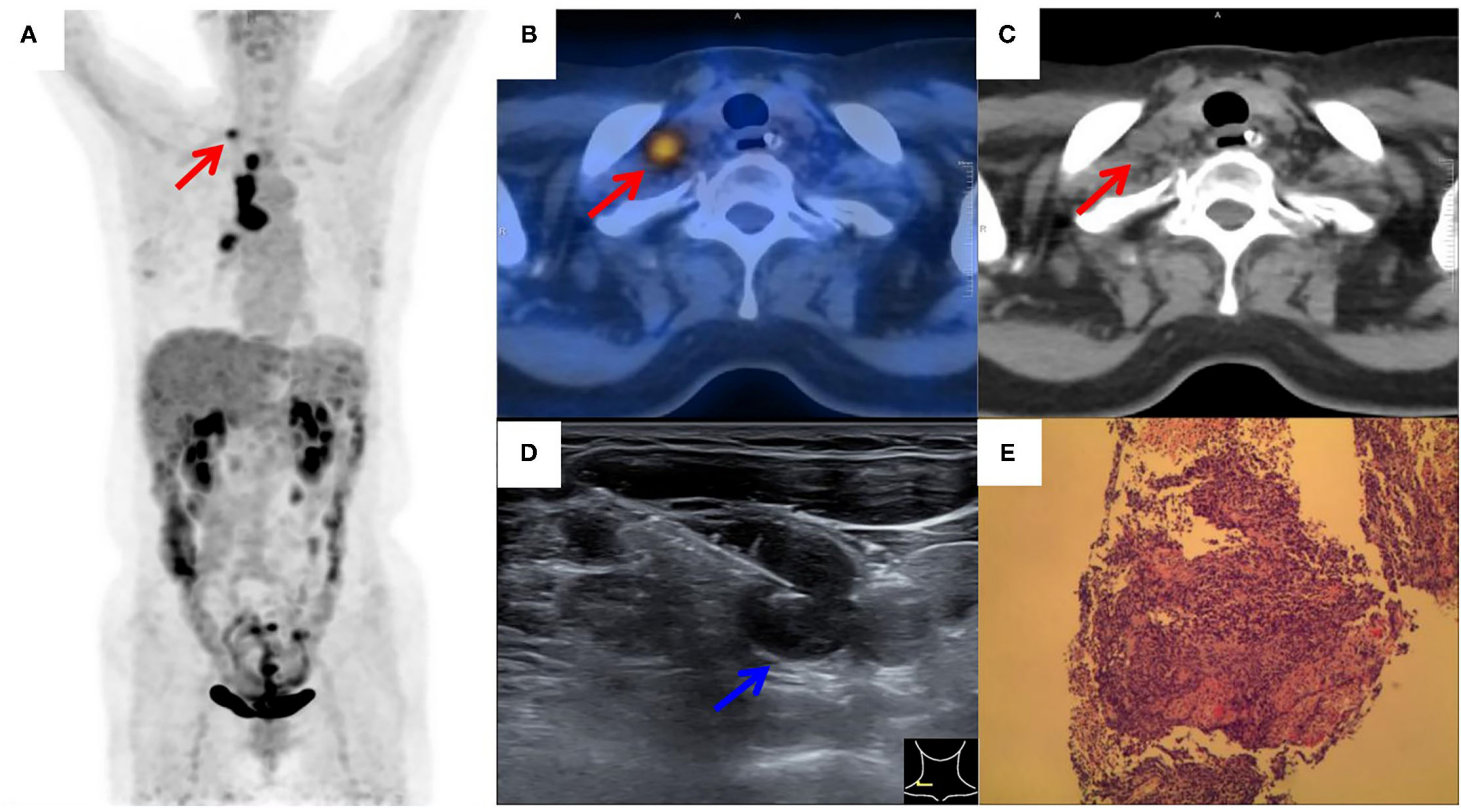
^18^F-FDG PET/CT of a patient with suspected lung cancer. **(A)** MIP image showed primary lung cancer in the right hilum with extensive hilar and right supraclavicular (red arrow) tracer accumulation; **(B,C)** axial PET/CT image showed FDG-avid SLN (red arrow) on the left side; **(D)** by consulting side-by-side PET/CT image, FDG-avid SLN was successfully located by the needle (blue arrow) under real-time ultrasound guidance; **(E)** a small cell lung carcinoma was histopathologically diagnosed.

Figures 2, 3 showed the examples of two suspected lung cancer patients who had successfully completed UGCB driven by FDG-avid SLNs.

## Discussion

Our retrospective analysis demonstrated a diagnostic yield of 98.1% (53/54) and a malignancy rate of 96.2% (51/53) for UGCB driven by FDG-avid SLNs in a cohort of patients with suspected lung cancer. Due to the superficial location, precise ultrasound guidance as well as the side-by-side PET/CT information, large SLNs specimens can be easily acquired by nuclear medicine physicians without complications.

The performance was higher than that of a previous report, in which pulmonologists-performed UGCB obtained a diagnostic yield of 95.7% (71/74) and a malignancy rate of 90.1% (64/71). However, among the 67 SLNs biopsied in their study, only 44 were FDG-driven and no specified method was described as to how PET/CT information was utilized during the sampling process (15). Two reasons might be considered to explain the higher performance in our study. Firstly, although both studies lacked well-defined criteria for SLNs selection, nuclear medicine physicians, who have more experience in PET/CT image interpretation, are more likely to choose SLNs with a high pre-test probability of success and malignancy. This might lead to a higher post-test diagnostic yield and malignant rate. Secondly, nuclear medicine physicians may be more focused on ensuring the needle placement toward the metabolically active area during the process of UGCB, thus pronouncing the benefit of FDG avidity in terms of diagnostic yield.

All 54 patients underwent UGCB with the aim of pathological diagnosis in the present study. Among them, fifty were confirmed as lung cancer by UGCB. In 28 patients, the confirmation of lung cancer in SLNs could define the disease stage (IIIB). For the remaining 22 patients, due to the existence of not-easy-to-access suspicious distant metastases on PET/CT (patients with easy-to-access suspicious distant metastases were excluded), exact pathological disease stage still could not be established. Although the guidelines recommended sampling of the lesions that would establish the highest stage of lung cancer, some patients may be more readily to accept a fast, simple and less painful procedure such as UCGB, which serves with high performance in pathological diagnosis and proving a non-resectable tumor stage (4, 5). In our department, the determination of the biopsy site was made considering multiple factors such as accessibility, risks involved with the procedures, and patients' preference.

Molecular analysis including EGFR mutation, ALK translocation and PD-L1 expression was adequately performed on the specimens obtained in the present study. Importantly, two SLNs samples with a diameter smaller than 10 mm successfully yield molecular testing results. This may indicate the powerful yield of UCGB in small lesions when PET/CT information was combined. In the patients tested for PD-L1 expression, 28.9% had a TPS of ≥ 50%, which was an indication for first-line immunotherapy in metastatic NSCLC (16). However, it should be noted that further studies are still needed to ultimately validate the guiding value of molecular information derived from metastatic SLNs on the survival benefits of NSCLC patients. For example, PD-L1 expression has been found to vary between different regions even within the same tumor, and previous studies found that PD-L1 expression in lymph nodes may not be a biomarker for treatment efficacy (17, 18).

Logistic advantages to the diagnostic pathway of suspected lung cancer patients can be expected from this technique. In our study, as a first-step after the PET/CT scan, UGCB driven by FDG-avid SLNs obliviated further interventional procedures in 38 patients in whom bronchoscopy, CT-guided lung biopsy, and diagnostic surgery had been planned. This may optimize the diagnostic process, especially in older patients who are with poor overall status and often have difficulties in complying with more complex and invasive diagnostic procedures. Moreover, the presentation of a PET/CT report, together with a pathological confirmation of stage IIIB disease, could swiftly exclude the patients from surgical therapeutic options and transfer them to oncology department. In this way, patients could have ample time to prepare for anti-tumor drugs while waiting for the molecular testing results if requested.

Several researchers reported that routine US evaluation of supraclavicular region and US-guided biopsy of suspicious SLNs in suspected lung cancer patients also have a high performance both for pathological confirmation and staging (19–21). In the present study, the US was solely used for guidance and localization, but not for selection of targeted SLNs, because we believed that FDG-avid SLNs would have a high probability for lung cancer metastases. To date, most of the investigations focusing on the performance of these two strategies were with small sample size and there exist no direct comparison studies as far as we know. Nevertheless, the high performance yielded from the present study verified our hypothesis and may initially justify the routine implementation of this PET-based selection strategy in suspected lung cancer patients who have already underwent ^18^F-FDG-PET/CT.

Major limitations including retrospective design, single center, small sample size and the lack of well-defined inclusion criteria may limit external validity of our findings. In this regard, well-designed studies are still needed to confirmed the high performance as well as the real added value of PET/CT information to this procedure.

## Conclusion

Nuclear medicine physicians-performed UGCB driven by FDG-avid SLNs in suspected lung cancer patients could produce a high performance in terms of diagnostic yield, malignancy rate, and molecular analysis, which may obliviate more invasive interventional procedures and lead to fast decisions on subsequent management.

## Data Availability Statement

The raw data supporting the conclusions of this article will be made available by the authors, without undue reservation.

## Ethics Statement

The studies involving human participants were reviewed and approved by the Ethics Committee of China-Japan Union Hosptal of Jilin University. The patients/participants provided their written informed consent to participate in this study.

## Author Contributions

SG and BJ conceived and design the study, which were proofed by SG. TW, JL, NL, SX, and LB collected and analyzed the data. TW and JL wrote the manuscript. All authors read and approved the manuscript.

## Funding

This research was supported by Research Fund of Science and Technology Department of Jilin Province (No. 20200201414JC) and Hygiene Specific Subjects of Jilin Province (Nos. 2019SCZ016, 2020SCZ12, and 2021SCZ05).

## Conflict of Interest

The authors declare that the research was conducted in the absence of any commercial or financial relationships that could be construed as a potential conflict of interest.

## Publisher's Note

All claims expressed in this article are solely those of the authors and do not necessarily represent those of their affiliated organizations, or those of the publisher, the editors and the reviewers. Any product that may be evaluated in this article, or claim that may be made by its manufacturer, is not guaranteed or endorsed by the publisher.
